# Barriers to health care access and utilization among aged indigents under the Livelihood Empowerment Against Poverty Programme (LEAP): the perspective of users and service providers in north-western Ghana

**DOI:** 10.1017/S1463423623000385

**Published:** 2023-07-24

**Authors:** Maximillian Kolbe Domapielle, Cornelius Dassah, Felix Dordaa, Benjamin Spears Ngmekpele Cheabu, Mohammed Sulemana

**Affiliations:** 1 Department of Governance and Development Management, Faculty of Public Policy, and Governance, Simon Diedong Dombo University of Business and Integrated Development Studies, Wa, U.W.R, Ghana; 2 The West African Center for Sustainable Rural Transformation (WAC-SRT), Simon Diedong Dombo University of Business and Integrated Development Studies (UBIDS), Wa, U.W.R, Ghana; 3 Department of Community Development, Faculty of Planning and Land Management, Simon Diedong Dombo University of Business and Integrated Development Studies, Wa, U.W.R, Ghana; 4 Christian Health Association of Ghana (CHAG), HIV/TB Community Systems Strengthening Program, Accra, Ghana; 5 Faculty of Health Science, Health Quality Programs, Queen’s University, Kingston K7L3N6, Canada

**Keywords:** aged indigents, health care access barriers, LEAP, NHIS

## Abstract

**Aim:**

This article draws on the poverty and access to health care framework to explore the barriers to access and utilization of primary health care among aged indigents under the Livelihood Empowerment Against Poverty Programme (LEAP) in Ghana.

**Background:**

Although many developing countries have made progress in extending primary health care to their populations following the Alma-Ata Declaration of 1978, the establishment of the Millennium Development Goals, and the Sustainable Development Goals (SDGs), barriers remain pervasive, particularly among vulnerable population groups. Previous studies have hardly paid in-depth attention to this important indicator for measuring progress toward achieving SDG 3.

**Methodology:**

To this end, we conducted a case study of access to health care services and utilization among aged indigents enrolled on the LEAP programme in the Daffiama Bussie Issa District of the Upper West. We collected and analyzed qualitative data from indigents aged 65 years and above, health care providers, and staff of the LEAP and the National Health Insurance Scheme (NHIS).

**Findings:**

Our analysis found geographic inaccessibility of health care, high costs of drugs and related services, exclusion of essential services from NHIS benefits package, and irregular transfer of cash to negatively influence access and utilization of health care among aged LEAP beneficiaries in the district. In addition to the need to strengthen the economy, provide health infrastructure and human resources for health in rural areas, the government needs to review the beneficiaries’ bimonthly stipends to reflect the daily minimum wage, eliminate the delay in payments, and review the benefits package of the NHIS to include essential services and medical devices commonly used by aged people. Yet implementing these recommendations has affordability implications that require innovation to mobilize additional resources and create the desired fiscal space and institutions that can sustainably implement universal coverage programmes such as the LEAP.

## Introduction

The current and projected rise in aged population refreshes the call for the implementation of Universal Health Coverage (UHC), particularly in the global south. The United Nations observe that globally, the population of older persons is increasing both in numbers and as a share of the total. The share of the global population aged 65 years or above is projected to increase from 10% in 2022 to 16 in 2050. By 2050, the number of persons aged 65 years or more worldwide is projected to be more than twice the number of children under 5, and about the same as the number of children under age 12 (UN, 2022). Africa’s aged population was 3.5% of 1.2 billion in 2015 and is projected to rise to 6% of 2.5 billion by 2050 (Leeson, 2018). In Ghana, the population of peopled aged 65 years and above is 4.3% of the total population. The population of this group is higher (5.3%) in the Upper West region (north-western) where the study was conducted (GSS, 2021). These statistics have implications for planning and implementation of programmes that are geared toward the achievement of universal health care and SDG 3, particularly in the global south where the current access levels of primary health care remain low (Domapielle, 2021; Domapielle *et al.*, 2021; 2022; Sarkpoh and Domapielle, 2022). The need to implement policies and programs to meet the health care needs of the rising aged population is in consonance with the pledges of governments to implement the 2030 Agenda for Sustainable Development, which includes SDG3, ‘ensure healthy lives and promoting well-being for all at all ages’ (UN, 2015). In addition to the need to establish age friendly health systems in support of this agenda, studies have also shown that people living in countries that have achieved UHC are healthier and live longer than those living without it (Ranabhat *et al.*, 2018), and that this policy promotes inclusive and sustainable economic growth and development (Owusu, 2014; Tangcharoensathien *et al.*, 2015; WHO, 2017). On the contrary, out-of-pocket payment (OOP)^1^ for health in Low and Middle-Income Countries (LMIC) results in drastic reductions in access to and utilization of health care services (Akazili *et al.*, 2017; Fenny *et al.*, 2018; Navarrete *et al.*, 2019). In these settings a significant proportion of the population is poor and cannot afford to pay out-of-pocket for health care services (Fosu, 2017; World Bank, 2018). Thus, the OOP system creates inequities in financial access to health care services in which poor individuals and households regularly postpone medical treatment, resort to self-medication, or rely on cheap quack practitioners, often with potentially harmful consequences (Boom *et al.*, 2004; Mensah *et al.*, 2010; Oppong, 2018). Reliance on OOP for health left close to half the world’s population lacking access to essential health services. As of 2017, the total global population facing catastrophic or impoverishing health spending was estimated to be in the region of 1.4 billion to 1.9 billion. From these statistics the population pushed into extreme poverty (at PPP$1.90 per day) was concentrated in low and lower middle-income countries (WHO, 2021).

Mindful of the need to reverse the financial difficulties imposed on the population by OOP, the government of Ghana introduced the National Health Insurance Scheme (NHIS) in 2003 through the passage of Act 650 of 2003 and the National Health Insurance Regulations of 2004 (L.I. 1809). The immediate objective is to increase financial access to quality primary health care by removing barriers such as out-of-pocket payments at the point of service use and gradually transition towards UHC by 2030 (UN, 2015). The scheme has a benefits package that covers about 95% of all disease conditions that affect the Ghanaian population (see Table 1), and members can access primary health care services under the package from any accredited health facility free of charge (NHIA, 2012b). Enrolment in the scheme has been mildly progressive. Active membership increased from 12.3 million in 2019 to over 16.8 million in 2020, representing 52% of the total population. From this enrolled population, the exempt category includes indigents (8%), Social Security and National Insurance Trust (SSNIT) contributors (4%), and SSNIT pensioners (1%). The rest are persons below 18 years (42%), persons aged 70 years and above (4%), and pregnant women (5%). The informal sector contributors, who constitute the only fee-paying NHIS membership category, constitute 36% of the active membership (NHIA, 2020). Research has shown that increased enrolments in the scheme have had a commensurate increase in utilization and improved health outcomes (Blanchet *et al.*, 2012; NHIA, 2012a; Van Der Wielen *et al.*, 2018).

**Table 1.** Benefits package of the NHIS

Outpatient Service	Inpatient Services
Consultations including reviews: these include both general and specialist consultations. Requested investigations (including laboratory investigations, X-rays, ultrasound, etc) for general and specialist out-patient services. Medication (prescription drugs on National Health Insurance Scheme Drugs List, traditional medicines approved by Food and Drugs Board and prescribed by accredited practitioners) Out-patients/Day surgical operations. (e.g., hernia repair, incision, and drainage, etc.) Out-patient physiotherapy.	General and specialist in-patient care Requested investigations (including laboratory investigations, X-rays, ultrasound scanning, etc.) for in-patient care Medication (prescription drugs on National Health Insurance Scheme Drug List, blood, and blood products) Cervical and breast cancer treatment Surgical operations In-patient physiotherapy Accommodation (General Ward) Feeding (where available).
3. Oral Health Services	4. Eye Care Services
Pain relief (e.g., incision and drainage, tooth extraction, and temporary relief) Dental restoration (simple amalgam filling and temporary dressing)	Refraction Visual fields A-scan Keratometry Cataract removal Eye lid surgery
5. Maternity Care	6. Emergencies
Antenatal care Deliveries (normal and assisted) Cesarean section Postnatal care	All emergencies shall be covered. These refer to crisis health situations that demand urgent intervention. They shall include: Medical emergencies Surgical emergencies (including brain surgery due to accidents) Pediatric emergencies Obstetric and gynecological emergencies (including cesarean section) Road traffic accidents Dialysis for acute renal failure
7. Public Health Services Funded Under Special Programmes	
Immunization Family planning In-patient and out-patient treatment of mental illness Treatment of tuberculosis, onchocerciasis, buruli ulcer, trachoma Confirmatory HIV test for AIDS patients	

NHIA (2012b).

A significant pro-poor component of the NHIS is the exemption specific categories of vulnerable groups of people from paying the membership fee. Included in the list are beneficiaries of the Livelihood Empowerment Against Poverty (LEAP) [see Table 2]. Like other exempt groups, enrolees of the programme are entitled to the NHIS’s full benefits package without the need to pay the membership fee (MGCSP, 2022).

**Table 2.** Exempt population groups

Pregnant women **Indigents, including LEAP beneficiaries.** Categories of differently abled persons determined by the Minister responsible for Social Welfare Persons with mental disorder Persons above seventy years of age (the elderly) Other categories prescribed by the Minister

NHIA (2012b).

The LEAP is a social protection programme implemented by the Government of Ghana (GOG) since 2008 to empower beneficiaries to eventually ‘LEAP’ out of extreme poverty. The categories of eligible members include orphaned and vulnerable children (OVC), persons with severe disability and elderly persons who are 65 years and above without productive capacity. Funds for the programme are drawn from the Government of Ghana (50%), donations from the Department for International Development (DFID) and a loan from the World Bank. These funds are used to provide bimonthly cash transfers and health insurance and other complementary services to beneficiaries across the country to alleviate short-term poverty and encourage long-term human capital development (Handa *et al.*, 2013). In the specific case of cash transfers, individual beneficiaries are paid a bimonthly stipend of GHS^2^ 64.00, 2-member households are paid GHS 76.00, 3-member households are paid GHS 88.00, and GHS106.00 for a household of 4 or more members (LEAP, 2022). Additionally, all LEAP beneficiaries are entitled to the full benefits package of the NHIS. Since inception, the programme has served as an important conduit through which its members enjoy financial risk protection against the costs of health care services (ILO, 2014; Agyemang-Duah *et al.*, 2019). A related study found that the LEAP 1000 cash transfer programme implemented in 10 districts in northern Ghana increased both emotional and instrumental support for pregnant women and mothers of children under one year living in poverty (de Milliano *et al.*, 2021). Similarly, LEAP beneficiaries reported that the cash transferred to them had improved their material and psychological well-being, increased food security and nutrition and removed the financial risk against the costs of primary health care (Alatinga *et al.*, 2020).

That notwithstanding, scholars have questioned the assumption that mere incorporation of fee payment exemptions for vulnerable groups such as the LEAP members guarantees them unfettered access to the benefits package of the NHIS (Domapielle *et al.*, 2020; Domapielle, 2021; Domapielle *et al.*, 2021). There is no doubt that increased enrolments in the NHIS have had a commensurate increase in utilization and improved health outcomes (Blanchet *et al.*, 2012; NHIA, 2012a; Van Der Wielen *et al.*, 2018); yet, there is no consensus on whether the poor have equal access to these benefits as the rich do. Besides, the removal of financial barriers at the point of service does not automatically guarantee the poor access to health care, as barriers such as distance to health facilities and travel related costs that might prevent vulnerable population groups such as indigents, aged, people with disabilities and the rural dwellers from accessing health care services even when they are insured under the NHIS still exist. For example, Akweongo *et al.* (2022) observed in their study of enrolment of indigents into the national health insurance scheme in Ghana through the LEAP in the Greater Accra, Brong-Ahafo and Northern region, that enrolment was low because of perceived poor quality of care, out-of-pocket payment, and long travel distance to service centers. Similarly, studies on the contribution of the LEAP programme to social inclusion in the Cape Coast Metropolis in the Central Region and the Shai Osudoku District in the Greater Accra Region found that, although the programme has contributed in alleviating poverty among vulnerable groups the stipends being transferred to beneficiaries were no longer realistic and therefore recommended an increase that will serve the needs of the beneficiaries (Agyemang *et al.*, 2014; Sackey, 2019). The objective of this study therefore is to explore whether aged indigents under the Livelihood Empowerment Against Poverty Programme (LEAP) who are enrolled under the NHIS are able to access primary health care when they need it. This is important because for many years, the issue of fiscal space for universal coverage programmes has been the subject of intense debate in various areas of public policy in developing countries. Whereas some analysts argue that universal coverage is not affordable for developing countries and should be postponed until they achieve developed country status, the recent global policy agenda for financing the implementation of the Sustainable Development Goals (SDGs), has sparked renewed interest in the problem of generating fiscal space to finance policies linked to the SDGs. The findings of this study lay bare the barriers faced by aged LEAP members in their quest to access and utilize health care services. Additionally, the findings contribute to the debate and analysis of the capacities of developing countries to create the desired fiscal space and institutions to sustainably implement universal health coverage and achieve SDG 3.

## Theoretical framework for assessing poverty and access to health care services

Research has established that the populations of developing countries tend to have less access to health services than those in developed countries, and within these developing countries, the poor have less access to health services than the rich (Narayan-Parker and Patel, 2000; Wagstaff, 2002; Domapielle, 2014; Domapielle, 2021; Domapielle *et al.*, 2022). Although limited access to health services, or the lack of it, is associated with barriers to accessing services, the correlation between poverty and lack of access to health services is also strong. For example, when health care is needed but is delayed or not obtained, users’ health condition is likely to deteriorate, which in turn results in lost income and higher health care costs, both of which contribute to poverty (Smith, 1999; Narayan-Parker and Patel, 2000). Thus, the relationship between poverty and access to health care can be analyzed as part of a larger spiral, where poverty results in ill health and ill health maintains poverty (Wagstaff, 2002). Although a comprehensive discussion of the meaning of poverty is not the focus of this article, we draw on the recognition of Peters *et al.* (2008) of poverty as extending beyond the concept of deprivation of income or material assets, along with Sen’s incorporation of the lack of freedom to lead the life people have reason to value (Sen, 2001). This implies that when people and communities are empowered to lead healthy lives they can overcome poverty (Stern *et al.*, 2006). Sen makes the point that relative income is important because it translates into capabilities, which is an important factor in accessing health services (Sen, 1995). Irrespective of the angle from which poverty is defined, there is a general consensus that it is associated with a lack of, or limited opportunity to access health care (Whitehead, 1991; Wagstaff, 2001). The World Health Organization (WHO) views access to health care as the continuing and organized supply of care that is geographically, financially, culturally, and functionally appropriate and adequate in content and in amount to satisfy the essential health needs of the users, and provided by methods acceptable to them (WHO, 1978). Consistent with this view, scholars who have critically analyzed the concept of access to health care services appear to concur that it is a multidimensional concept that ought to be disaggregated into a set of operational dimensions that can be assigned specific indicators to make it easy to understand. These operational dimensions include geographic accessibility, affordability, availability, and acceptability of health care services (Penchansky and Thomas, 1981; Peters *et al.*, 2008; McIntyre *et al.*, 2009). In this section, the operational dimensions of poverty and access to health care services in low-income settings are reviewed as the underpinning framework for discussing the results of the study.

Considering the typical challenge of limited resources for health in developing countries, an assessment of the geographic dimension of access to health care is critical in the discourse around the barriers and facilitators of uptake among aged users. Geographic access is the relationship between the location of health care facilities and the location of those who need it, taking account of users’ transportation resources and travel time, distance and cost (Aday and Andersen, 1974; Penchansky and Thomas, 1981; Peters *et al.*, 2008; McIntyre *et al.*, 2009). According to McIntyre *et al.* (2009), it involves issues such as the relationship between the location of health care facilities and the location of users and their transportation opportunities. In the provision of optometric care, for example, the question will be: are optometric services located and configured in ways that reflect the variations in need for these services in the population? This is an important part of accessing health care in developing countries considering that health facilities are thinly spread across the country and populations in rural settlements may be compelled to travel a long distance to access health services (Matthew, 1973; Aday and Andersen, 1974; Aday and Andersen, 1981; Khan *et al.*, 2005; Peters *et al.*, 2008; Jacobs *et al.*, 2012; Macha *et al.*, 2012). In urban areas where health infrastructure is robust, geographical distance may be less of a barrier to access to care, and subsequent determinant of health outcomes (Mathews *et al.*, 2010). However, where service provision is sparse, transport infrastructure is weak, and populations are predominantly poor, distance often leads to delays in deciding to seek care, not reaching the right health facility and not receiving the needed care (Buor, 2003; Campbell *et al.*, 2006; Gabrysch *et al.*, 2011). This study attempts to establish whether distance to health services (both NHIS offices and health facilities) poses a barrier to access to health services among aged members of the LEAP programme or not.

Availability of services is another important determinant of access to health care. Availability in the framework of access to health services means having the right type of health care available to those who need it. This would include such things as hours of operation and waiting times that meet demands of those who would use care, as well as having the appropriate type of service providers and materials (Penchansky and Thomas, 1981; Peters *et al.*, 2008). An important availability issue highlighted in the literature is the ability and willingness of service providers to serve the population in accordance with the type and severity of their condition (McIntyre *et al.*, 2009). Aside from these, the relationship between the type, range, quantity, and quality of health care services provided at a facility and the nature and extent of the health needs of the individuals being served, equally determine service availability. For example, are laboratory services adequate and satisfactory? Do facilities provide comprehensive care or does comprehensive care require referrals between different facilities in multiple locations? In a study by Dussault and Franceschini (2006), they found that irrespective of income status, all countries studied reported a higher proportion of health personnel in urban and well-endowed areas. The access literature as it relates to availability of health services in Ghana focuses almost exclusively on the shortage and uneven distribution of health facility personnel between and within regions (Durairaj *et al.*, 2010; Apoya and Marriott, 2011; Jehu-Appiah *et al.*, 2011; Schieber *et al.*, 2012; Atinga, 2012; MoH, 2014; Atinga *et al.*, 2015).

Affordability has always been at the center of the debate on health care accessibility especially for the poor. It refers to the relationship of prices of health care services and users’ ability to pay in the context of the household budget and the other demands on the budget (Penchansky and Thomas, 1981; Peters *et al.*, 2008; McIntyre *et al.*, 2009). As already highlighted, besides the direct costs of treatments and paying for drugs, there are also indirect costs that deter the poor from seeking treatment when they need it (Macha *et al.*, 2012; Mills *et al.*, 2012; Averill and Marriott, 2013; WHO, 2015). These indirect costs include the opportunity costs of time of both the patients and household members accompanying them, transportation costs, and expenses on food and lodging (Penchansky and Thomas, 1981; Macha *et al.*, 2012; McIntyre *et al.*, 2013; Kusi *et al.*, 2015; WHO, 2015).

Ability to pay relates to an individual’s ability to secure funds from their household or family, and the other demands placed on those potential sources of funds (Jutting, 2001; Jütting, 2003; ILO, 2008; McIntyre *et al.*, 2009). These include for instance, the eligibility of individuals to benefit from financial support from publicly funded health care financing schemes that subsidize or cover the costs of health care at the time-of-service use. Ability to pay also includes the ability of households or family units to cover the costs of services at the point of delivery, including: the amount, timing, and frequency of income flows, and the individual’s ability to draw on these income streams; the level of cash savings that can be used to cover health care costs; the assets owned by the household and whether these assets can be easily and rapidly translated into cash; the extent and nature of social networks from which households can mobilize cash either via gifts or loans; the ability to secure formal credit arrangements; and the conditions for loans (McIntyre *et al.*, 2009).

Acceptable health services provision remains a major challenge in low-income countries although the Declaration of Alma-Ata (WHO, 1978) proposed that primary health care needed to be in line with prevailing cultural norms (Peters *et al.*, 2008; Dillip *et al.*, 2012). Acceptability relates to the fit between provider and patient attitudes and expectations of each other (Penchansky and Thomas, 1981; Gilson *et al.*, 2007; Peters *et al.*, 2008; McIntyre *et al.*, 2009). Acceptability is also defined as the social and cultural distance between health care systems and their users (Hausmann-Muela *et al.*, 2003). Acceptability barriers come in different forms depending on the setting. In European settings, such barriers are identified as underpinning the systematic differences in health care utilization patterns that exist between socioeconomic groups and between other population groups (Tamsma and Berman, 2004). In low- and middle-income countries, the focus is on poor provider–patient relationships, an element of the social and cultural distance is often raised as an important access barrier (Palmer, 2007).

**Figure 1.**
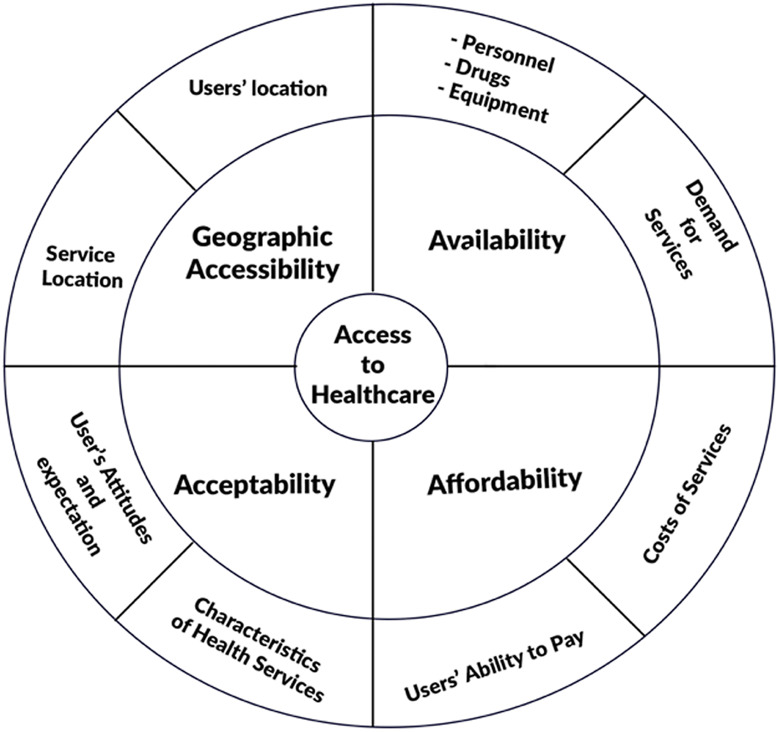
Conceptual model for assessing poverty and access to health care. Based on Peters *et al.* (2008)

Gilson *et al.* (2007) observe that the most important element of acceptability is the cultural competence of health systems, and they argue that cultural incompetence is frequently demonstrated by the dissonance between health beliefs of patients and dominant medical knowledge, discrimination toward patients, communication barriers between patients and providers, and mistrust of health providers. In tackling health inequity, therefore, it is important to recognize the socialized nature of health care. According to Gilson *et al.* (2007), tackling acceptability and trust barriers calls for three sets of actions: actions that strengthen the provision of care to the benefit of all groups while offering particular gains to disadvantaged groups including rural populations; actions that prioritize the health care needs of disadvantaged groups; and actions that are necessary to enable and sustain the interventions. Collectively, these policy actions can strengthen universal health care systems to benefit disadvantaged groups (Fig. 1).

## Methodology

### Study context

**Figure 2.**
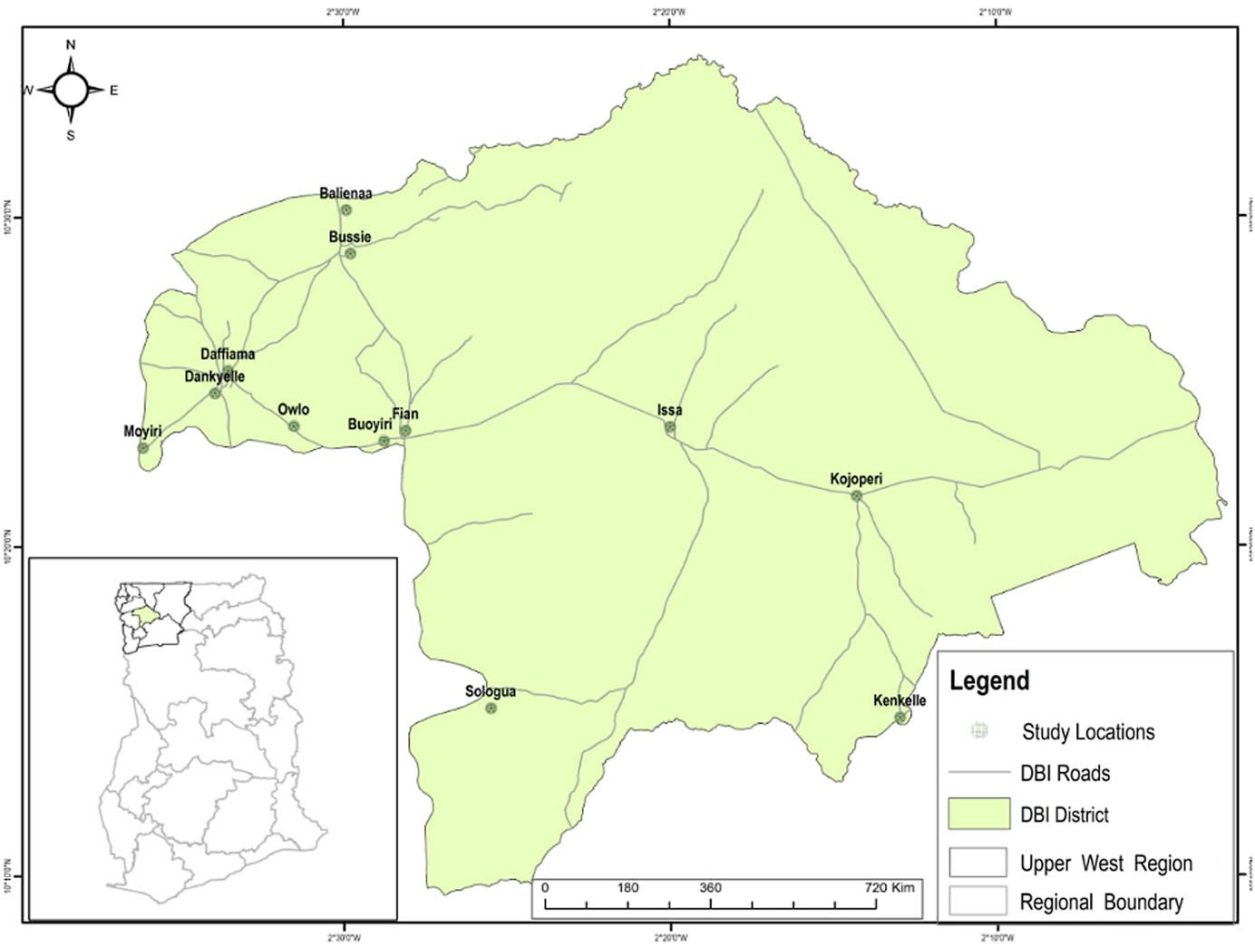
Map of Daffiama-Bussie-Issa district

The study was carried out in the Daffiama-Bussie-Issa District in the Upper West Region of Ghana (see Fig. 2). The Upper West, within which the Daffiama-Bussie-Issa District (DBI) is located is the poorest region in Ghana. It has a staggering 66% of the population considered as extremely poor, 18% as poor and only 16% as non-poor (GNHR, 2021).^3^ The proportion of the population aged 65 years and above is 5.3%, which is 1% higher than the national average of 4.3% (GSS, 2021).^4^ Located within a semi-arid climatic zone, the region experiences a single maximum rainy season between April and September. This is followed by a prolonged period of drought, as a result of the influence of the dry and hazy north easterly Harmattan winds. The DBI was carved out of the Nadowli district in 2012 by the District Assemblies’ Law 1988 under legislative instrument (LI) 2104 with Issa as its capital. It is bounded by Sissala West District to North, Wa East District to the East, Wa Municipal to the South, and Nadowli-Kaleo to the West. The district has a total population of 38 754, comprising 19 831 (51.2%) females and 18 923 (48.2%) males. It is largely rural as 33 508 (86.5%) and 5,246 (13.5%) of the population reside in rural and urban areas, respectively. The main income-generating activity of residents is seasonal subsistence agriculture, and the main farm crops include cowpea, groundnuts, maize, and millet. Crop yield is low due to overreliance on rainfall, poor soil fertility, primitive farming practices, and limited use of technology (GSS, 2021). This results in low productivity and spiraling poverty in the region, a finding made by previous research (GSS, 2014, 2018; Cooke *et al.*, 2016; Akurugu *et al.*, 2021). This context of the limited livelihood options and the pervasiveness of poverty in the study area illuminates the potential of the LEAP programme to ameliorate poverty and provide financial risk protection against the cost of illness for the beneficiary indigents. Given the relationship between aging and vulnerability to diseases, the latter is of added value.

Currently, the district has thirteen (13) health facilities comprising one (1) polyclinic at Issa and four (4) health centers. Although there is an ambulance to facilitate the movement of emergency and referral cases to higher level facilities for treatment, the district does not have a hospital and the average distance to these 13 health facilities is 9 km, which exceeds the national target of 5 km (GSS, 2014). This understanding of the inadequacy of health resources in the district is important for analyzing and identifying the barriers and enablers of utilization and access to health care among poor aged populations in rural settings.

### Study design

The study employed an exploratory case study of access to health care services and utilization among indigents enrolled on the LEAP programme in the Daffiama Bussie Issa District of the Upper West Region. This design made it possible to intensively explore for the specific barriers to health care access among aged LEAP beneficiaries in the district. The process involved a review of relevant literature on social protection for the aged generally, universal health coverage, the LEAP and NHIS programmes in Ghana, and health care access and utilization. We then collected primary data through semi-structured in-depth interviews with diverse study participants and gained a deeper understanding of the barriers and enablers of health care use among aged indigents under the LEAP.

### Sampling strategy

The multistage sampling approach was employed in the selection of the district, communities, and participants. The Daffiama-Bussie-Issa district was selected for the study not just because it is one of the poorest districts, it is also the only district in the region without a hospital. The absence of the district hospital has implications for access to referral services and the general quality of health care. The selection of communities and health facilities was informed primarily by enrolment in the LEAP programme; thus, only communities enrolled in the LEAP programme were selected. Twelve (12) out of the thirty-five (35) LEAP communities in the district were purposively selected. The selection of these 12 communities was informed by the zonal delineation of the district into DBI North, DBI West, and DBI East. Thus, four communities (4) were selected from each of the three zones. Of these four (4) communities two (2) have health centers and two (2) are without health centers. The rationale for this variation is to enable us to compare the extent of access and or utilization of health care services among aged LEAP enrollees in communities with health centers and those without them. In DBI West, the communities with health facilities are Daffiama and Owlo, and those without them are Dankyelle and Buoyiri. In DBI North, Bussie and Fian have health facilities, while Moyiri and Balienaa are without health facilities. Finally, in DBI East, whereas Issa and Kojokpere have health facilities, and Kenkelle and Sologuo do not have health facilities.

**Figure 3.**
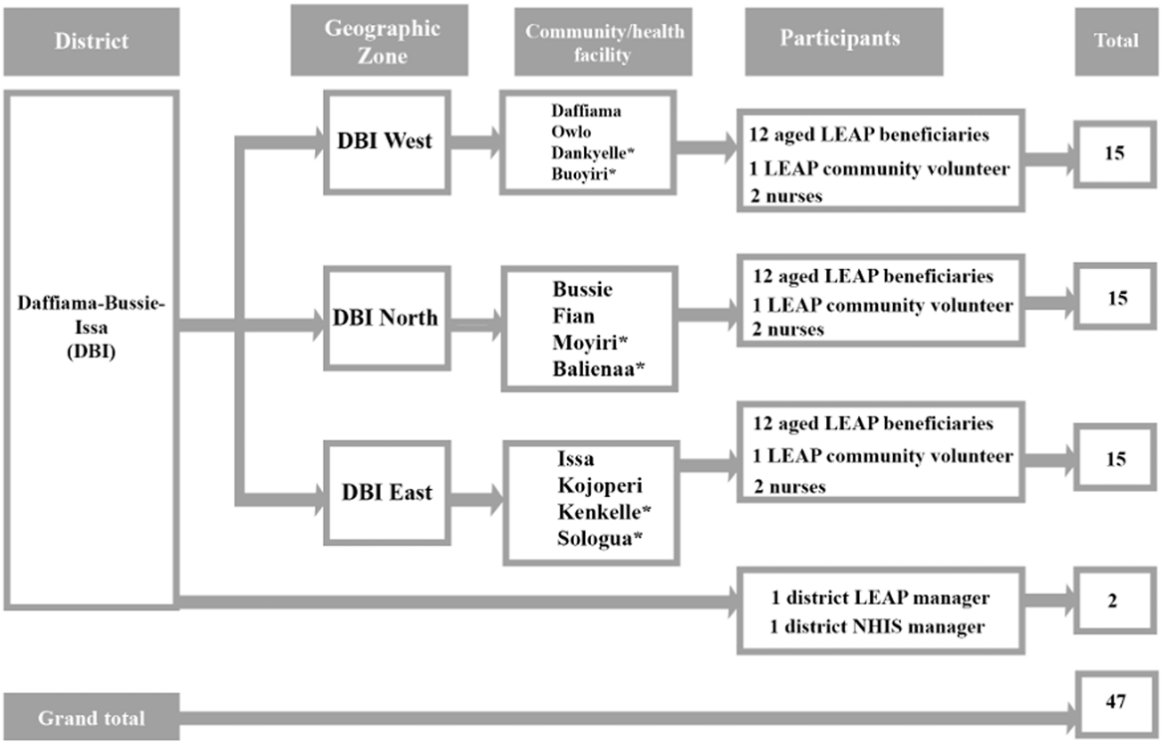
Multistage sampling strategy

The selection of participants was done in three phases. We initiated the process by selecting the District LEAP Officer and District NHIS Manager. The District LEAP Officer provided the list of LEAP Community Volunteers in the district from which we purposively selected three of them, one from each of the three zones. These volunteers facilitated the convenient selection of three (3) aged LEAP beneficiaries each from the four (4) communities in their zones. The LEAP beneficiaries who participated in this study were included because they had fallen sick and visited or attempted visiting a primary health facility in the previous 12 months before the study. Figure 3 illustrates the multistage sampling strategy employed for the study. The communities without health facilities are assigned an asterisk mark (*).

### Data collection

This study was conducted between September 2020 and December 2022. Semi-structured in-depth interviews were used to generate data for the study. Using semi-structured interview guides, we conducted in-depth interviews with the District Managers of the LEAP and NHIS programmes. These two key informants provided information on the benefits that aged LEAP enrollees are entitled to under the NHIS and the challenges faced by aged LEAP enrollees in renewing their membership of the NHIS to guarantee them free access to primary health care services when they need them. We then proceeded to interview the LEAP Community Volunteers, who, in addition to answering questions on the challenges associated with enrolling and renewing the NHIS membership of aged LEAP beneficiaries, provided information on their location. The last group of participants interviewed for this study were the aged LEAP enrollees. They provided information on the benefits that accrue to them, and the challenges associated with accessing health care services; they also proffered some recommendations for health policy and planning. The interviews were conducted in English for interviewees who could speak it, and in Dagaare (the local language) for users who do not speak English. All the interviews were audio-recorded for transcription and analysis.

### Data processing and analysis

We employed the thematic analytical framework to analyze the data and identify barriers associated with aged LEAP enrollees’ utilization of primary health care services in the district. We initiated the analyses by transcribing the audio recorded interviews. To get familiar with the data, we read each interview transcript line by line, noting down repetitions, commonalities, and disparities that are relevant to the research question. In the margins of each page, we wrote down the main themes from the page’s conversation as preliminary analysis. We then examined the themes a second time and then organized them along thematic networks. In the concluding phase, we used the soft copies of the transcripts to pull together the portions of data that represented each theme and developed the qualitative analysis by analyzing in detail the responses of the district Managers and the LEAP and NHIS programmes, Nurses, LEAP Community Volunteers, and LEAP enrollees in relation to these themes and ultimately the research question.

### Ethical issues

Important ethical issues, including anonymity, confidentiality, and consent, were given premium in this study. Informed consent was obtained from all participants before each interview was conducted. Participants were made aware that their decision to participate in the study was purely voluntary, and they could withdraw from the study at any time or skip any question(s) they did not wish to answer. All the information provided by the participants is protected and treated as strictly confidential.

## Results

This section provides results on the participants included in the study as well as results related to the barriers to health care access among aged indigents under the LEAP. These include high costs of transport, drugs and related services, the exclusion of essential services from NHIS benefits package, and inherent challenges with the cash transfer component of the LEAP.

Table 3 shows that the study participants were between the ages of 65 and 90. Most of them were in the age cohort of 65 and 69 (41.7%), followed by those aged 70 to 74 (25%), and those aged 75 to 79 (22.3%). Participants aged 80 years and more constituted only 11.1% of the total sample of LEAP beneficiaries. In terms of the gender composition of the sample, 55.6% are females and 44.4% of them are males. This is consistent with the gender structure of the population of the district which has 51.2% females and 48.2% males. The total sample size of 36 LEAP beneficiaries was evenly districted among the 12 study communities. Most of the participants (52.8%) did not have formal education, 27.8% had primary school education, 16.7% had secondary school education, and only 2.8% of them had tertiary education. These figures have implications for access to health-related information among the aged population. On marital status, most of the participants were married (77.8%) and 22.2% were widowed. In terms of the duration of their LEAP membership status, 66.7% of them had benefited for the past three years or more, and 33.3% of them had benefited for the past two years. On the issue of annual cash transfers received, we found that all of them had received GHS 256.00 in the past 12 months in 2021 instead of GHS 384.00. This means that two cycle’s payment cycles were outstanding. Compared with the country’s 2023 daily minimum wage set at GHS14.88, the cash transferred to LEAP beneficiaries appears to be insufficient to pay for transportation and other health care access-related charges. Lastly, most of the participants (77.8%) were active members of the NHIS and 22.2% inactive members, and their visit to health facilities for treatment varied from weekly (5.6) to never (8.3%). The ensuing subsections present the results on the barriers to health care access among aged indigents under the LEAP. Verbatim quotes from participants are drawn upon to support the views expressed by the participants.

**Table 3.** Demographic profile of the LEAP beneficiaries interviewed

Variable	Category	*n* = 36	Percentage
Age	65-69	15	41.7
	70-74	9	25
	75-70	8	22.2
	80+	4	11.1
Gender	Male	16	44.4
	Female	20	55.6
Community	Daffiama	3	8.33
	Owlo	3	8.33
	Dankyelle	3	8.33
	Buoyiri	3	8.33
	Bussie	3	8.33
	Fian	3	8.33
	Moyiri	3	8.33
	Balannia	3	8.33
	Issa	3	8.33
	Kojokpere	3	8.33
	Kenkelle	3	8.33
	Sulaguo	3	8.33
Level of education	No formal education	19	52.8
	Primary education	10	27.8
	Secondary education	6	16.7
	Tertiary education	1	2.8
Marital status	Married	28	77.8
	Never married	0	0
	Widowed	8	22.2
	Divorced	0	0
Duration of LEAP membership	1 year	0	0
	2 years	12	33.3
	3 years+	24	66.7
Cash transfer received in 2021	GHS 64	0	0
	GHS 128	0	0
	GHS 192	0	0
	GHS 256	36	100
	GHS 320	0	0
	GHS 384	0	0
NHIS membership status	Active^5^	28	77.8
	Inactive^6^	8	22.2
Preferred primary source of health care	Orthodox health facility	21	58.3
	Traditional medicine provider	15	41.7
Frequency of health facility visit	Daily	0	0
	Weekly	2	5.6
	Monthly	6	16.7
	Quarterly	9	25
	Half-yearly	10	27.8
	Yearly	6	16.7
	Never	3	8.3

^5^Active members of the NHIS. These are beneficiaries who are enrolled in the scheme and their membership status is active.

^6^Inactive members are the beneficiaries who are enrolled in the scheme, but their membership status is active because they have not renewed their membership.

### Geographic accessibility of health care

This is associated with diverse factors including long distances between users’ residences and the health facilities nearest to them, bad roads linking the communities, and the limited availability of ambulance service. In terms of the distance to health facilities, the only polyclinic and the four (4) health centres in the district are sited in towns, so rural residents are required to travel long distances to access these facilities. All the aged LEAP beneficiaries in this study blame long distance to health facilities as impeding access to and utilization of health care services. The following interview extracts sum up aged LEAP members’ perception of the geographic accessibility of health care:

*‘When you are referred to a higher facility, transportation becomes a problem. If you do not have money for transport, you have to walk a distance close to 10 kilometres. I am unable to do that distance at age 72’* (An interview with a 72-year-old man at Buoyiri).

Similarly, an aged LEAP beneficiary from Daffiama with whom we interacted expressed concern about the distance she frequenctly covers in order to access health referral care:

*‘For the transportation issue, I don’t want to talk about it, but I have a problem with my back, around my spinal cord and because of that, I visit the Nadowli hospital about 2-3 times a month and sometimes I get referred to Nandom to meet a specialist. The road from here to Nadowli, and to Nandom is very bad yet I sit on the motorbike to be transported to these facilities. I have delayed and missed a number of hospital appointments in the past because of the unbearable fatigue involved in traveling long distances to health facilities.’* (An interview with a 67-year-old woman at Daffiama).

Some of the nurses interviewed for this study admitted that the distances between users’ homes and the health facilities are unfavorable and therefore discourage regular visits to the health facilities even though they are NHIS members. A nurse at the Issa Polyclinic with whom we interacted had this to say about distance and poor visits to facilities by the aged:

*‘Most of the aged people in this area delay in coming for attention. They come only after they have tried a number of herbal treatments without success. And, I think the long distance to this facility accounts for the delay.’*

While also admitting that long distance to health facilities deters some aged users from visiting the facilities when they are sick, some of the nurses are of the view that aged people have a strong preference for herbal medicine, which is the reason they delay in seeking modern health care. A nurse at the Bussie Health Centre said this in connection with aged people’s preference for traditional medicine:

*‘Yes, distance is a barrier but do not forget that most of these old people prefer to report their condition initially to their village tradtional medicine practitioner for attention, and only turn to us when there is no improvement.’*

A combination of long distance and bad roads to health facilities has made transportation by public passenger transport unaffordable to many including poor aged people. The majority of them have had to rely on motorbikes and tricycles to physically acccess health centers when they fall ill. These alternative means of transport are not only unsafe to use but they are also uncomfortable and end up worsening their health condition. An aged LEAP beneficiary lamented the fatigue she normally endures when transported on a motor bike to the health center at Bussie:

*‘No passenger vehicle comes to this village so my newphew would occasionally offer to carry me on his motrobike to the health centre at Bussie for treatment when I am sick. The challenge however is that because of the bad state of the road I would return feeling worse than I was prior to the trip.’* (An interview with a 76 year-old LEAP beneficiary at Balania).

One would have expected the Ghana government’s One District-One Ambulance project to address the transportation challenge faced by rural dwellers in accessing health care, but that has not happened so far. The ensuing excerpts reflect the perspective of the health care providers on how the poor transportation system impedes aged people’s utilization of health care services:

*‘Transportation is a challenge for them. Those in the distant communities find it very difficult coming to the centre. They always want to come but the means of transport becomes a problem. Such people only come to the facility when they can no longer handle their condition.’* (An interview with a Nurse at the Issa Polyclinic).

Similarly, a nurse said this, in connection with the poor transportation situation in the district:

*‘Transportation is a challenge for most healthcare users, particularly aged people needing health care. Most of them walk long distances to come and access healthcare and sometimes, their condition deteriorates. These challenges keep them from reporting early and when their conditions get worse, we are required to detain them, and that further increases their woes.’* **(**An interview with a Nurse at the Daffiama Health Centre).

A community health nurse at Issa Polyclinic summed up the transportation situation in the district as follows:

*‘The biggest challenge impeding the accessibility to health services is how to get to the health centers. If we make means of transportation available at the CHPS compound, it will help them a lot. Due to the lack of family support, some of them cannot transport themselves and as a result, they delay in seeking health care until their condition gets worse.’* (An interview with a Community Health Nurse at Issa Polyclinic).

The results show that although some aged LEAP beneficiaries prefer to use the services of traditional health practitioners, there is consensus among the participants that long distances between users’ homes and the health facilities nearest to them, bad roads linking the communities, and the limited availability of ambulance service are significant barriers in the utilization of health care. Besides these, however, costs associated with the purchase of drugs, feeding, and lodging also emerged as important barriers in the utilization of health care among aged LEAP members.

### Costs associated with the purchase of drugs and other services

The results show that the costs of drugs, feeding, and lodging when seeking health care are often not affordable to aged LEAP beneficiaries. A 75-year-old man from Issa explained his routine struggles with paying for in-patient care and related services:

*‘I frequently visit the health centre because I am suffering from high blood pressure which I need to check regularly. Occasionally, when my blood pressure is very high, they would detain me for observation for several days. Because the NHIS benefits package does not cover all the drugs prescribed to me which puts me in a difficult financial situation.’*

Similarly, an aged LEAP beneficiary explained the many occasions she could not afford the cost of drugs prescribed to her to buy at the pharmacy shop:

*‘Buying drugs is a huge burden for me. Most of the time, there are no drugs at the health centre so they would write the prescription for me to go and buy. The little stipend I receive from the LEAP programme is spent on drugs.’* (An interview with a 67-year-old woman at Kenkelle).

Other older LEAP beneficiaries shared their experiences of catastrophic spending on drugs, even though such drugs are covered by the NHIS. One of them had this to say on the subject:

*‘After meeting with the nurse, she usually writes medicines for me to collect from the pharmacy. However, often, the pharmacy does not have those drugs so they will also write another piece of paper for me to go and buy my drugs. So, I spend more money on the drugs even though those drugs are covered by the NHIS.’* (An interview with a 70-year-old man in Sulaguo).

Another aged LEAP beneficiary said this:

*‘The only thing I spend my money on is medicines. There would not be a problem if I could afford the medicines prescribed for me to buy at the pharmacy. Unfortunately, I am poor and as a result I am not able to pay if the drugs are not available at the facility and free of charge.’* (An interview with a 67-year-old man at Fian).

Our interaction with health care providers confirmed that contrary to the NHIS exemption policy, aged LEAP beneficiaries are paying out-of-pocket for essential drugs:

*‘Although most of the poor older people possess a valid NHIS card, they are sometimes required to pay for the drugs and services that are not included in the NHIS benefits package.’* (An interview with a Nurse at Daffiama Health Centre).

Another nurse asserted that:

*‘Those without NHIS pay for the consultation. However, those with NHIS cards do not pay for the consultation. They may only pay for drugs. Also, they spend money on laboratory testing.’* (An interview with a Nurse at the Issa Polyclinic).

Following these concerns, a nurse at the Bussie health center suggested a review of the benefits package for LEAP beneficiaries to make it possible for them to buy essential drugs and pay other medical bills at the health facilities:

*‘The LEAP should assist beneficiaries to pay for their hospital bills like helping pay for drugs or in case they are referred to a higher facility. Some of them struggle a lot when they are sick.’* (An interview with a Nurse at the Owlo CHPS).

These findings point to a high prevalence of out-of-pocket payment for health care services by aged LEAP members contrary to the NHIS exemption policy that aged LEAP beneficiaries with valid NHIS cards are exempt from the payment of fees at the point of service use. Yet the evidence also speaks of the thinness of the bimonthly stipends paid to aged LEAP members. In the following section, we present results on the comprehensiveness of the NHIS benefits package.

### Comprehensiveness of the NHIS benefits package

Results on the comprehensiveness of the NHIS benefits package were mixed. The LEAP beneficiaries, the district LEAP officer, and the nurses raised concerns about the range of services that are available in practice. The expectation is that a UHC package would include a comprehensive set of health services available in the right quality and quantity, and the delivery would be in harmony with the cultural values and sensitivities of the users. A participant from Dankyelle said this in connection with the comprehensiveness of the NHIS benefits package:

*‘Even when I go with my National Health Insurance card, I am still asked to pay for drugs. Often, we do not have money. The last time I visited the hospital, they wrote drugs for me to go and buy but because I do not have money, I couldn’t buy them. Now I am treating myself with traditional medicine*.’ (An interview with a 70-year-old woman, Daffiama).

Similarly, an aged LEAP beneficiary from Owlo was unimpressed about the range of services available to her as a NHIS card bearer. The following is her response when asked about the comprehensiveness of the NHIS benefits package.

*‘They have never given me drugs at the hospital even though I go with my valid NHIS card. They always prescribe the drugs for me to go and buy. We the older people, our diseases are many and we expect to be treated for free because we are poor. I do not even know the drugs that are covered by the NHIS. The only thing I benefit with this [NHIS] card is free consultation.’* (An interview with a 78-year-old poor older woman at Owlo).

These views were corroborated by health care providers. A nurse at the Issa Polyclinic had this to say in connection with the comprehensiveness of the NHIS benefits package;

*‘The payments they make with the NHIS are mostly for drugs and laboratory services. Some medicines are not covered by the NHIS and hence they must pay for them. Because of this most of them do not seek healthcare promptly yet and this category of people have a weak immune system and cannot withstand sicknesses. I suggest the hospitals should have social welfare services to cater for the needs of those who do not have NHIS cards’* (An interview with a Nursing Officer at the Issa Polyclinic).

However, while conceding that the NHIS has some implementation lapses the district Manager of the scheme disagreed with the view that the benefits package is limited. He argued that the scheme has one of the most generous benefits packages among countries with similar economic characteristics. He said:

*‘Aside the NHIS in Ghana, I am not aware of any developing country that is implementing a health insurance scheme with a benefits package covering 95% of all disease conditions and provides free maternal care and has exemptions for vulnerable population roups. The range of services covered by the scheme is indeed comprehensive.’*

On his part, the district LEAP Officer observed some shortcomings on the part of both the NHIS and the aged LEAP beneficiaries. While agreeing with the NHIS Manager that the scheme’s benefits package covers a comprehensive range of services, he was quick to add that certain essential services such as ambulance service and medical devices such as hearing aids, medicated glasses, and dentures commonly used by vulnerable aged people are excluded from the package. Other services in the package (seen in Table 1) are often not available at the point of use. The main shortcoming on the part of the aged LEAP beneficiaries is the failure to regularly renew their membership of the NHIS. Although the registration is free of charge enrollees are still required to renew their membership cards once every year. Unfortunately, many of them do not adhere to this rule and when they visit the health facility without a valid health insurance card, health providers are unable to serve them free of charge.

### Challenges with the cash transfer scheme

The transfer of cash to beneficiaries was described by participants as insufficient, irregular, and sometimes inaccessible and impedes access to and utilization of health care. There was consensus among the participants that the currents stipends being paid to beneficiaries are woefully insufficient to pay for their basic needs. A beneficiary said this about the meagerness of the stipends:

*‘They have been paying this same amount for more than three years. It is too small to pay for the necessities such as food items, lorry fares to the health centre and drugs. The government should consider increasing it because the cost of living has become so high. (An interview with a LEAP beneficiary in Saluguo).*

Delay and irregular transfer of cash to beneficiaries also emerged strongly as a barrier to access to health care among this group of LEAP beneficiaries. The cash transfers are sometimes in arrears of up to two cycles (4 months), and during these periods most of them are unable to pay for transportation to a health facility when they fall sick. The following is a statement from a participant who missed her hospital appointments because her stipend did not arrive on time:

*‘I have missed my doctor’s appointment at the Nadowli hospital twice because the money did not come on schedule, and I did not have anyone to lend me money. They do this all the time, and it affects us so badly.’ (An interview with a LEAP beneficiary in Daffiama).*

The district LEAP office confirmed this concern when an Officer said this in an interview:

*‘They are right that the payments are not regular. Sometimes we owe them up to two cycles and even though the arrears are paid in the third cycle, we would prefer to pay them on schedule.’*

In summing up the results, we reiterate that the barriers to access and utilisation of health care among aged indigents in the study area include geographic inaccessibility of health care, high costs of drugs and related services, the exclusion of essential services from the NHIS benefits package and inherent challenges with the cash transfer component of the LEAP. These barriers are discussed critically in the next section of the article.

## Discussion

This study explored access to and actual utilization of health care services by aged LEAP beneficiaries in the DBI district and uncovered significant barriers. These include geographic inaccessibility of health care, high costs of drugs and related services, the exclusion of essential services from the NHIS benefits package, and the delays that characterize the cash transfer scheme. This finding has implications for Ghana and other developing countries aiming to achieve SDG 3 by 2030.

These barriers are discussed in turn, starting with geographic inaccessibility of health care, where the participants concurred that long distances between users’ homes and the health facilities nearest to them, bad roads linking the communities, and the limited availability of vehicular transport work against their ability to physically access health facilities in the district. Given the significance of geographic accessibility within the poverty and access to health care framework (Peters *et al.*, 2008), several other empirical and theoretical studies have paid considerable attention to it and have observed in the context of developing countries that health facilities are often spread thinly and populations in rural settlements are often compelled to travel a long distance in search of treatment (Matthew, 1973, Aday and Andersen, 1974; 1981; Khan *et al.*, 2005; Jacobs *et al.*, 2012; Macha *et al.*, 2012; Domapielle *et al.*, 2020). In urban areas where health infrastructure is relatively robust, physical distance may be less of a barrier to accessing health care and less a subsequent determinant of health outcomes (Campbell *et al.*, 2006; Mathews *et al.*, 2010; Domapielle *et al.*, 2020). However, in DBI where 86.6% of the population reside in rural communities and are mostly poor, where service provision is thin, transport infrastructure is weak, and populations are predominantly poor, it is no surprise to find aged LEAP beneficiaries not seeking health care promptly, failing to reach the right health facility or preferring the services of traditional medicine practitioners. This finding is important for policy because, first, research has shown aged populations are more susceptible to illness because of their age, low-income status, and sometimes location (Bhat and Kumar, 2017; Gabrani *et al.*, 2021). Second, because the NHIS has a pro-poor mandate, there is the need to ensure equity in the distribution of the benefits. Equity in this regard would require the implementation of positive discrimination measures not just in the form of exemptions but also in the provision of health infrastructure and human resources for health, as well as improving the provision of outreach services in mobility-constrained communities. These measures are expected to improve the provision of primary health care to the aged who are unable to travel long distances to seek medical attention.

Similarly, high costs of drugs and related services emerged strongly as an impediment to the satisfactory utilization of health care among aged LEAP beneficiaries. An important policy concern is the high prevalence of out-of-pocket payment for health care services reported by aged LEAP beneficiaries, as this clearly contradicts the NHIS exemption policy that aged LEAP beneficiaries with valid NHIS cards are exempt from the payment of fees at the point of service use. Although out-of-pocket payment for health care in the era of the NHIS has been previously reported (Dalinjong and Laar, 2012; Akazili *et al.*, 2014; Domapielle, 2021; Domapielle *et al.*, 2021; Domapielle *et al.*, 2022), this specific finding about LEAP beneficiaries paying out-of-pocket for health care refreshes the debate about the efficacy of the NHIS and whether the poor, for whom the scheme was established to provide financial health protection, are actually reaping the benefits. Besides the direct costs of paying for drugs and related services, indirect costs associated with expenses on food and lodging are found to deter poor aged LEAP beneficiaries from seeking health care. Again, while this finding is not new in the public health literature (Penchansky and Thomas, 1981; Macha *et al.*, 2012; McIntyre *et al.*, 2013; Kusi *et al.*, 2015; WHO, 2015), it raises concern about the adequacy of the bimonthly cash transfer package to LEAP beneficiaries. Contrary to previous research that associated improvement in the living conditions of the beneficiaries of the cash transfers and the free NHIS enrolment (Alatinga *et al.*, 2020; de Milliano *et al.*, 2021), we found that the bimonthly stipends transferred to the beneficiaries fall far below the country’s 2023 daily minimum wage of GHS 14.88. Coupled with the current cost of living crisis in the country, we associate with the position of the beneficiaries that the stipends they receive are insufficient to cater for their basic needs. In addition to the meagerness of the stipends, the transfers are irregular, and as observed in Table 3, each of the beneficiaries had been paid a total amount of GHS 256.00 as of December 2021 instead of the full annual stipend of GHS 384.00. This, as the results indicate, impacts significantly on utilization and access to health care services. This finding calls for measures to ensure the timely release and transfer of stipends to the beneficiaries, and a review of the LEAP programme to arrive at realistic stipends for beneficiaries taking into consideration the existing daily minimum wage and the current economic conditions in the country.

The range of services provided under the NHIS benefits package is up for scrutiny in this study. This is because, on paper, the benefits package covers about 95% of the burden of diseases (BoD) in Ghana (Witter and Garshong, 2009; Wang *et al.*, 2017). These benefits range from outpatient to public health services funded under special program (see Table 1). Whereas this package has been described as generous and satisfies an important objective of UHC, it has also attracted criticisms. The most immediate of these is the exclusion of primary health care services including ambulance service and medical devices like hearing aids, medicated glasses, and dentures, which are mostly used by the elderly population. The consequence of excluding these essential services is catastrophic health care spending and or constrained access to health care, which results in adverse health outcomes among the poor aged population. This ultimately raises concern about the scheme’s genuine commitment to health equity and Ghana’s objective of achieving UHC by 2030. To keep this important health objective on track an immediate measure would include a review of the NHIS benefits package to cover essential services and devices commonly used by aged populations. While ensuring equity in the distribution of the benefits of the scheme is important, it must not be done at the risk of the scheme’s financial sustainability. Critical fiscal space elements such as cost containment and adverse inclusion must guide decisions around a revised benefits package.

## Conclusions and implications for policy and practice

In wrapping up, the study found that geographic inaccessibility of health care, high costs of drugs and related services, exclusion of essential services from the NHIS benefits package, and delay in the payment of stipends to beneficiaries negatively influence the extent to which aged LEAP beneficiaries utilize primary health care services in the district. The consequences of these for the aged poor are delay in seeking medical treatment, self-medication, and or reliance on traditional medicines, all of which have potential adverse health outcomes and exacerbates poverty among this population group. While the findings lay bare the barriers faced by aged LEAP beneficiaries in the utilization of health care, they also trigger a fresh debate and analysis of the capacities of developing countries to create the desired fiscal space and institutions to sustainably implement social protection schemes including universal health coverage, and for that matter, the achievement of SGD 3. Any innovative approaches to address these barriers would have fiscal space implications. For example, our recommendations include the provision of additional health infrastructure and human resources for health in deprived districts, the provision of outreach services in communities that are mobility constrained, a review of the cash transfer program to eliminate delay in payments, the use of the current daily minimum wage as a benchmark, and lastly, a review of the benefits package of the NHIS to cover a range of essential services and medical devices commonly used by aged people. Whereas these measures will make primary health care accessible to poor aged people and improve their health and well-being and ultimately contribute toward the achievement of SDG 3, our findings illuminate the need for further and deeper discourse of the fiscal space dilemma faced by developing countries such as Ghana in the quest to sustainably implement universal coverage programmes.

## Limitations of the study

By employing a qualitative approach, we were able to explore deeper for detail understanding and analysis of the barriers to access and utilization of health care among aged indigent LEAP beneficiaries, especially related to the study location. This notwithstanding, the study was limited to the DBI district in the Upper West region, and as such our findings reflect mainly the views of this limited group of participants and cannot be generalized to reflect the barriers to access and utilization of health care faced by all indigents under the LEAP programme in Ghana.

## Footnotes

1 Out-of-pocket payments (OOP) for health are direct payments made to health care providers by individuals at the time of services use (WHO, 2010).

2 GHS is the currency code of the Ghanaian Cedi. The Ghanian cedi is the official currency of Ghana.

3 GNHR stands for Ghana National Household Register.

4 GSS stands for Ghana Statistical Service.
